# Book Review: Pandemics, Politics, and Society. Critical Perspectives on the Covid-19 Crisis

**DOI:** 10.3389/fsoc.2021.699018

**Published:** 2021-05-26

**Authors:** William Outhwaite

**Affiliations:** Sociology, Newcastle University, Newcastle upon Tyne, United Kingdom

**Keywords:** pandemic, social transformation, science, technocracy, expertise

**A Book Review on**

**Pandemics, Politics, and Society: Critical Perspectives on the Covid-19 Crisis**

*by Gerard Delanty (Berlin: De Gruyter), 2021, 215 pages, ISBN: 978-3110713237.*

The pandemic has made amateur epidemiologists and, later, vaccinologists of us all. Social science has been more in the background: in the United Kingdom, for example, a group of social and behavioural scientists was called on by government mainly to advise about the prospects of public compliance with restrictive measures. Gerard Delanty has however risen to the challenge of confronting the questions which the pandemic poses to social and political theory, initially with a journal article (Delanty, 2020) and now with this major and very impressive edited volume, which brings together some of the world’s leading sociologists.

As Delanty notes (p. vii) the pandemic is arguably the most significant social transformation since the end of Soviet communism. As with what Claus Offe called at that time a ‘triple transition’, there is now a triple crisis: ‘a health and medical crisis, an ecological one, and a crisis in capitalism and globalization.’ (p. 2) As with a Covid test, the pandemic has imposed an invasive and painful test of current practices of agriculture and globalised agribusiness and travel, and produced a positive, in other words a bad result. ‘The pandemic has become a metaphor for a flawed world’ (p. 19).

In a neat fit with the theme of this special section, Delanty suggests the possibility, based on the experience of earlier pandemics such as the Black Death and the 1918 flu, ‘that out of the current crisis will come some improvements in public policy and a more humanized kind of capitalism than the current precarity that predominates. But such gains took decades if not centuries…’ (p. 17).

The book is divided into three sections, on expertise, globalisation and the social. In the first, Claus Offe points to the different categories of people divided by the pandemic and the policies for its mitigation, focusing in particular on those who cannot afford to isolate and ‘are forced into a trade-off of life and livelihood.’ (p. 34) Something like the same dilemma confronts public authorities, though those which delay introducing restrictive measures end up having to make them more severe and prolonged. Stephen Turner, who has worked substantially on the politics of expertise, explores the paradoxes of its current politicisation, as do Jan Zielonka, drawing on recent analyses of the opposition between technocracy and populism and the emergent phenomenon of ‘technopopulism’ (Bickerton and Ivernizzi-Accetti, 2021), and Daniel Innerarity. Jonathan White, who has analysed the EU’s response to earlier emergencies, and writing before the vaccine supply cock-up of early 2021, examines the particular difficulties of a transnational response.

In the second part of the volume, the sociologist of science Helga Novotny points to the way the pandemic contributes to digitalisation and datafication, and Eva Horn to its implications for the ecological crisis of the anthropocene age. ‘If the pandemic can teach us a lesson for managing the future in the Anthropocene, it is not only about tipping points. It is also about the immense cost of dithering and of scepticism towards scientific findings.’ (p. 135) Bryan Turner also adopts a *longue durée* perspective on the ‘political theology of Covid-19’ and earlier pandemics, while Daniel Chernilo, like Frédéric Vandenberghe and Jean-François Véran, stress its global nature. When Marcel Mauss wrote about the ‘total social fact’ of the gift, he was not thinking of a gift (in the German sense of poison) affecting the whole world, and now even Antarctica, at more or less the same time. ‘Like the collars and shells of the Kula or the animal furs of the potlatch, it circulates freely within social relationships and brings into movement the whole of society…’ (p. 175) As Chernilo points out, ‘At its peak, lockdowns, quarantines, restrictions of travel, work and education reached around 80% of the world’s 7 billion of inhabitants.’ (p. 167) While Véran, who had worked with Médecins sans Frontières in Paris and contracted Covid, Vandenberghe, less dramatically, ‘endured the anguish of social isolation. We were experiencing, perhaps, the inversion of Sartre’s formula: hell, after all, might be the absence of other people.’ (p. 186).

In the third and final part, Sylvia Walby shows how social democracy can mitigate the social consequences of the pandemic, and Donatella della Porta suggests that emergencies such as the current one can open up opportunities for social movements, as well as curtailing their activities. The political economist Sonja Avlijaš argues that, in a context where we are all more or less seriously threatened, ‘the coronavirus pandemic has made a big dent in the already weakened ideology that the “competitive society” does not need security and protection’ (p. 240). Albena Azmanova takes a similar line that the ‘battlegrounds of justice’ against inequality and catastrophic climate change now include a third battleground directed against precarity resulting from social polarisation: ‘the pandemic showed precarity to be the real grievance of the 99 per cent...’ (p. 254).

This wide-ranging book is a timely and extremely important contribution which will stand comparison with the host of subsequent books on the topic which we can expect to follow.
