# Erratum: Book review: Pandemics, politics, and society: Critical perspectives on the Covid-19 crisis

**DOI:** 10.3389/fsoc.2022.1089684

**Published:** 2022-11-22

**Authors:** Frontiers Production Office

**Affiliations:** Frontiers Media SA, Lausanne, Switzerland

**Keywords:** pandemic, social transformation, science, technocracy, expertise

Due to a production error, the citation for the book being reviewed was missing from the article. These details have now been inserted at the beginning of the article. The text that has been inserted is:

**“A Book Review on**

**Pandemics, politics, and society: Critical perspectives on the Covid-19 crisis**

by Gerard Delanty (Berlin: De Gruyter), 2021, 215 pages, ISBN: 978-3110713237.”

Due to a production error, the running title was incorrect. The running title has been updated to “Book Review: Pandemics, Politics and Society”.

The publisher apologizes for these mistakes. The original version of this article has been updated.

